# Supplementary material for: Metabolomics Reveals Tyrosine Kinase Inhibitor Resistance-Associated Metabolic Events in Human Metastatic Renal Cancer Cells
Source: Int J Mol Sci. 2024 Jun 7;25(12):6328. doi: 10.3390/ijms25126328 (PMC11204329; doi:10.3390/ijms25126328)
Supplement: Supplementary file 1 [file ijms-25-06328-s001.zip › ijms-3024274-supplementary.pdf]

Supplementary Materials

Metabolomics reveals tyrosine kinase inhibitor resistance-associated metabolic events in human metastatic renal cancer cells

Filipa Amaro[1,2,*], Márcia Carvalho[1,2,3,†], Maria de Lourdes Bastos[1,2], Paula Guedes de Pinho[1,2], Joana Pinto[1,2,*]

[1] Associate Laboratory i4HB-Institute for Health and Bioeconomy, University of Porto, 4050-313 Porto, Portugal

[2] UCIBIO-Applied Molecular Biosciences Unit, Laboratory of Toxicology, Department of Biological Sciences, Faculty of Pharmacy, University of Porto, 4050-313 Porto, Portugal

[3] RISE-UFP, Health Research Network, Faculty of Health Sciences, University Fernando Pessoa, 4200-150 Porto, Portugal

[†] Current address: LAQV/REQUIMTE-Associated Laboratory for Green Chemistry of the Network of Chemistry and Technology, University of Porto, Porto, Portugal.

*Corresponding authors
Filipa Amaro (F.A.), famaro@ff.up.pt
Joana Pinto (J.P.), jipinto@ff.up.pt

Address:
Associate Laboratory i4HB and UCIBIO
Laboratory of Toxicology, Department of Biological Sciences
Faculty of Pharmacy, University of Porto
Rua Jorge Viterbo Ferreira, 228
4050-313 Porto, Portugal
Tel.: +351 220428796

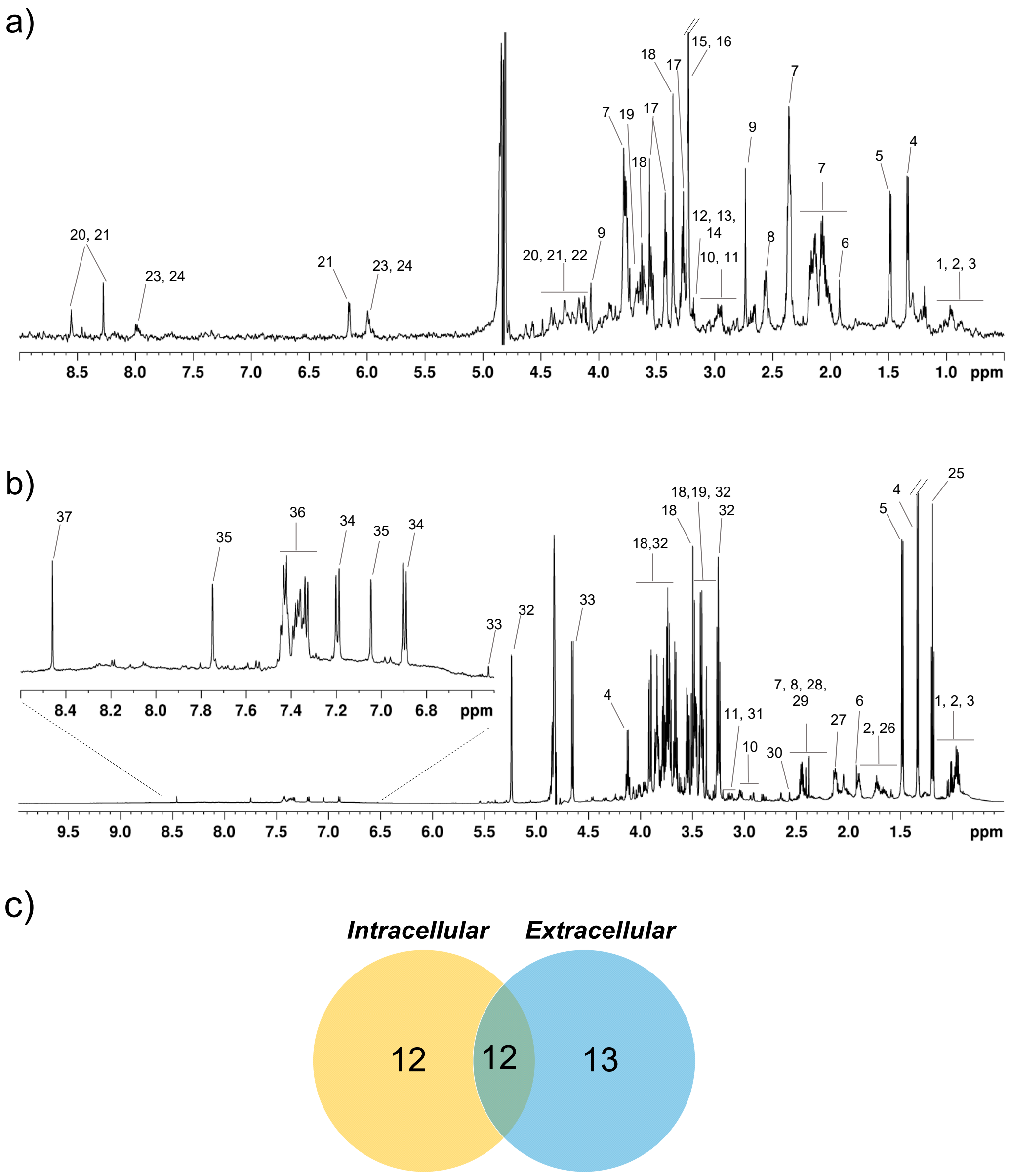

Figure S1. (a, b) Representative ^{1}H NMR spectra obtained for the intracellular polar extract and the extracellular culture medium of Caki-1 cells, respectively. (c) Venn diagram illustrating the number of unique and shared metabolites between the intracellular polar extract and the extracellular culture medium of Caki-1 cells. 1: Isoleucine; 2: leucine; 3: valine; 4: lactate; 5: alanine; 6: acetate; 7: glutamate; 8: glutamine: 9 glutathione; 10: aspartate; 11: asparagine; 12: creatine; 13: phosphocreatine; 14: ethanolamine; 15: o-phosphocholine; 16: glycerophosphocholine; 17: taurine; 18: myo-inositol; 19: glycine; 20: ADP; 21: ATP; 22: NAD+; 23: UDP-glucose, 24: UDP-galactose; 25: ethanol; 26: arginine; 27: methionine; 28: pyruvate; 29: succinate;; 30: pyroglutamate; 31: lysine; 32 glucose; 33: fumarate; 34: tyrosine, 35: 1-methylhistidine; 36: phenylalanine; 37: formate.

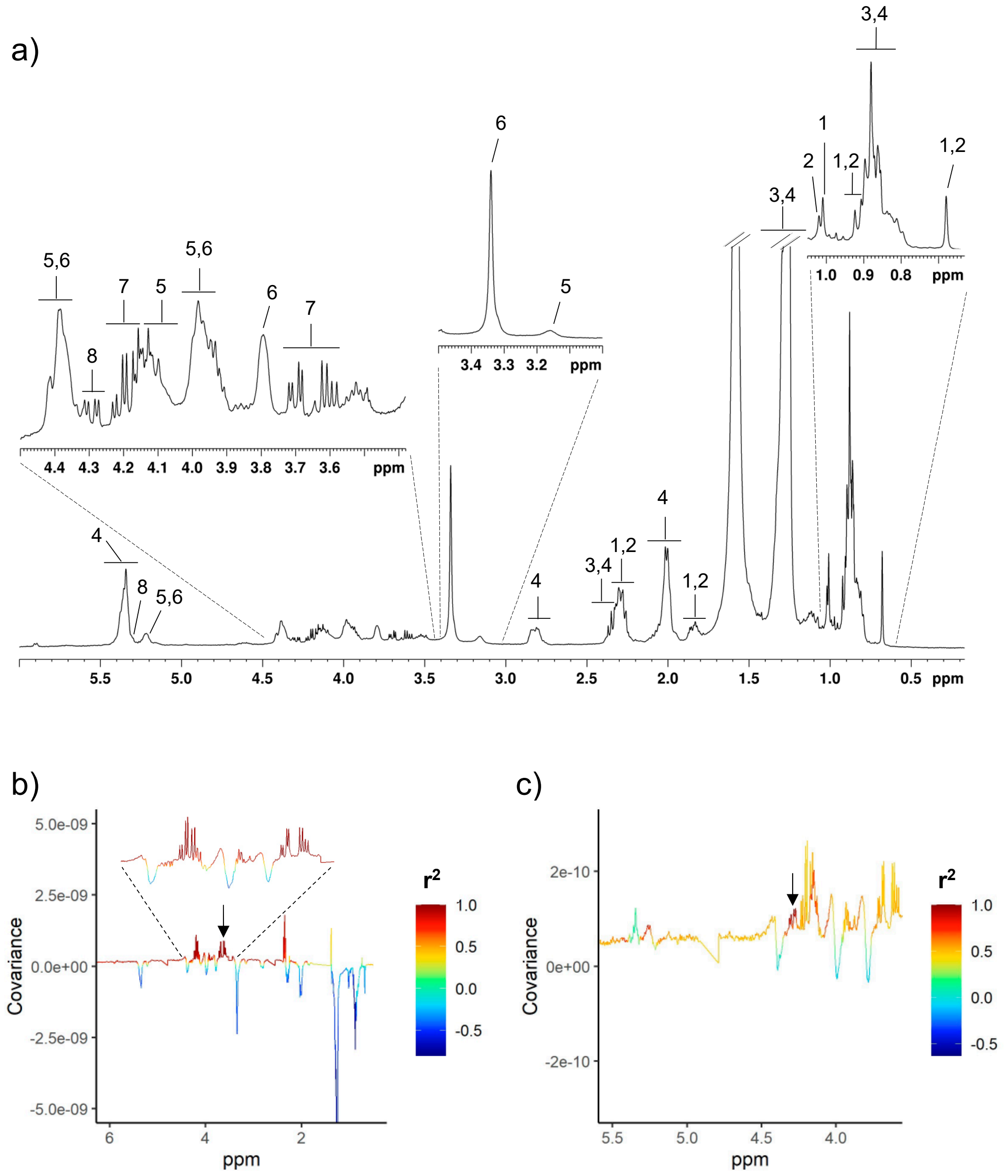

Figure S2. a) Representative ^{1}H NMR spectrum obtained for an intracellular lipid extract from Caki-1 cells. b) STOCSY spectrum of the intracellular lipid extract computed using the resonance at 3.62 ppm from monoglycerides as driver peak (black arrow). c) STOCSY spectrum of the intracellular lipid extract computed using the resonance at 4.28 ppm from triglycerides as the driver peak (black arrow). 1: cholesterol; 2: cholesteryl esters; 3: saturated fatty acids; 4: unsaturated fatty acids; 5: phosphatidylethanolamine; 6: phosphatidylcholine; 7: monoglycerides; 8: triglycerides.

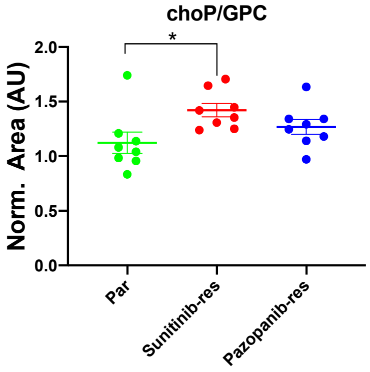

Figure S3. Boxplot of phosphocholine/glycerophosphocholine ratio (choP/GPC) for sunitinib- and pazopanib-resistant Caki-1 cell lines. The statistical significance was assessed by comparison with the parental Caki-1 (* p-value ≤ 0.05).

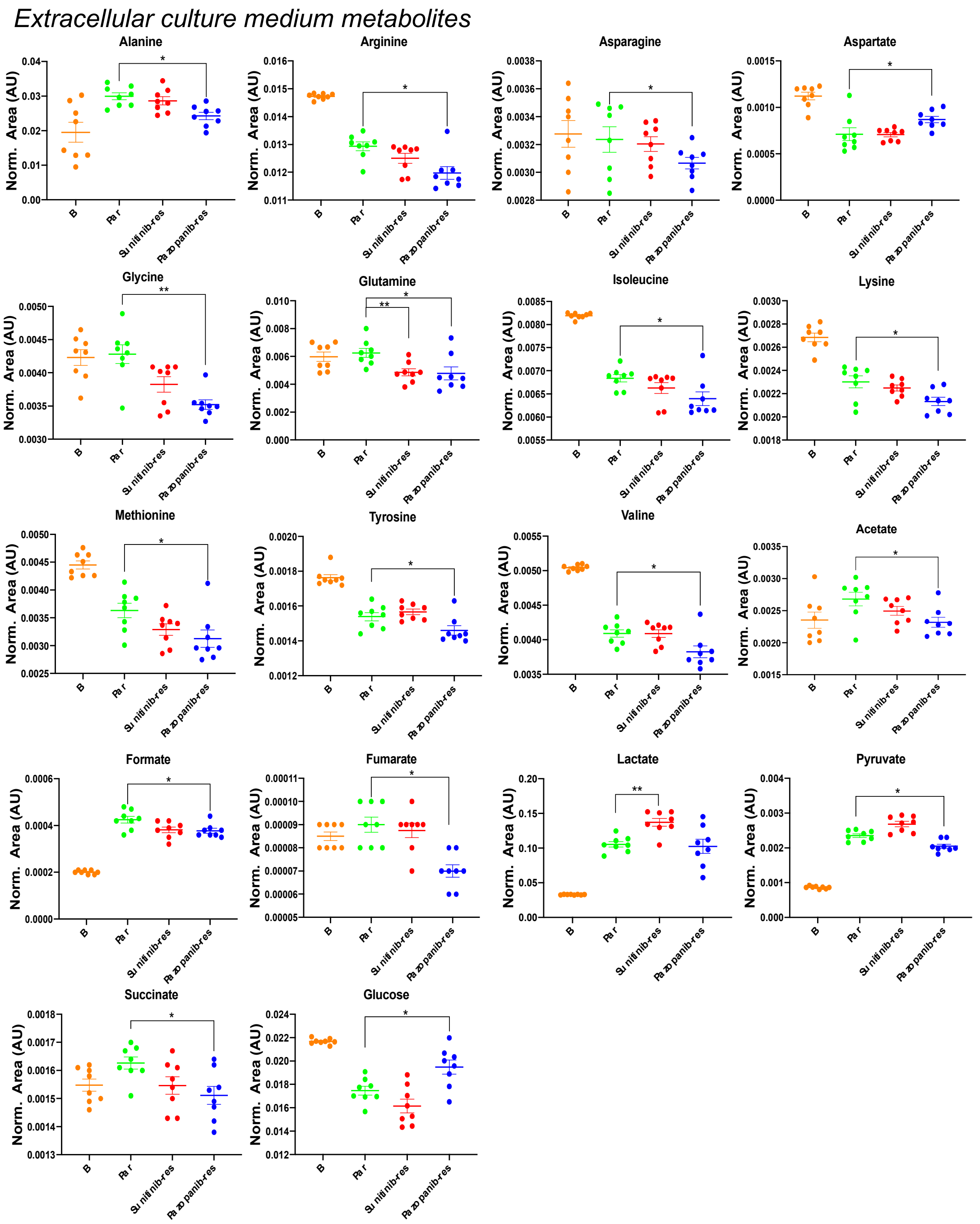

Figure S4. Boxplots of metabolites found to be significantly altered in the extracellular culture medium of sunitinib- and pazopanib-resistant Caki-1 cell lines. Statistical significance was assessed by comparison with the parental Caki-1 cell line (* p-value ≤ 0.05, ** p-value ≤ 0.01).

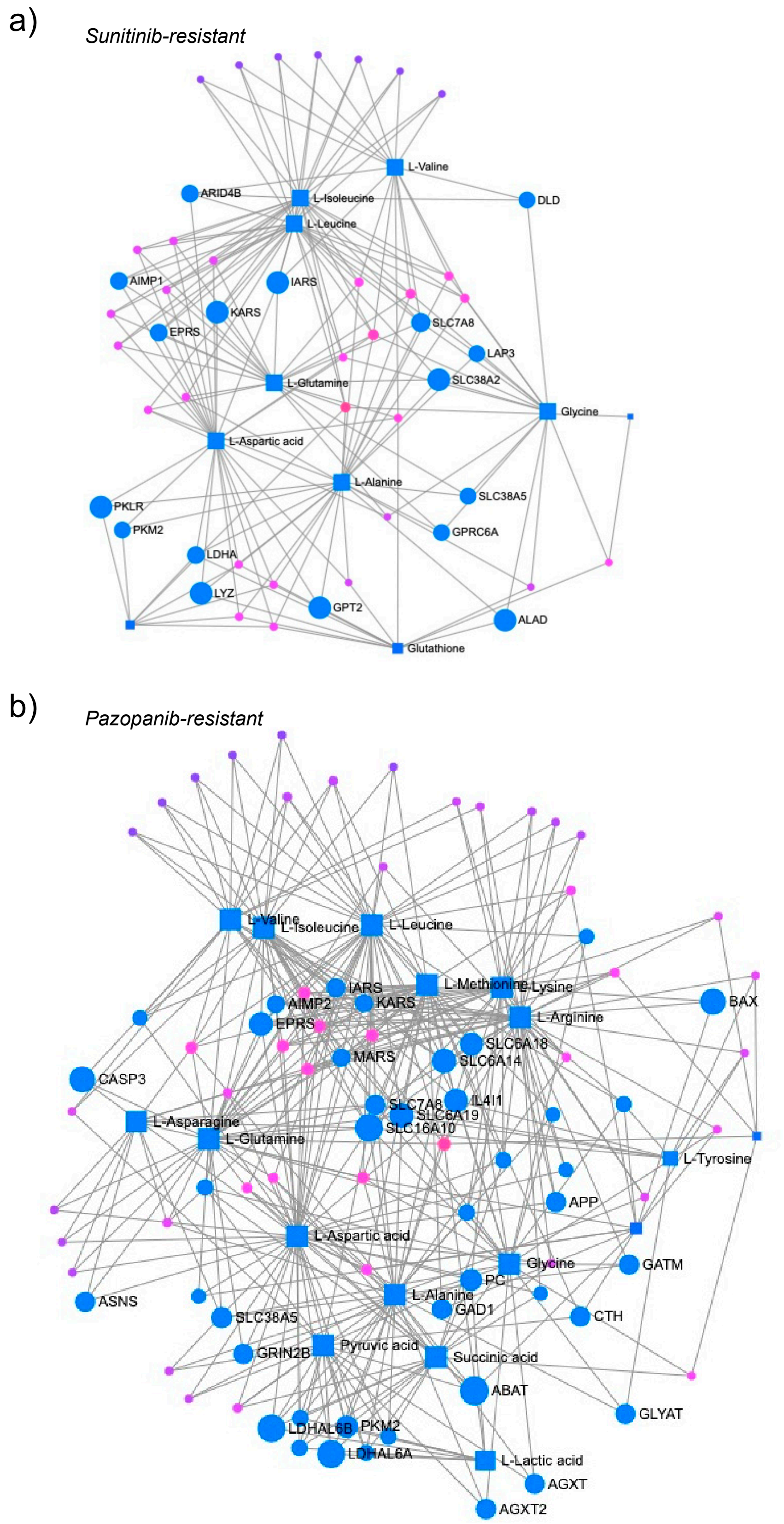

Figure S5. (a, b) Metabolite-gene network analysis showing the significantly altered metabolites (blue squares) and putative relative related-genes (blue circles) in sunitinib- and pazopanib-resistant Caki-1 cell lines. Node colours represent betweenness centrality scores, which reflect the centrality of a node in a

network (calculated by measuring the number of shortest paths through the node). Node size indicates node degree, which is the number of connections a node has to other nodes.

Table S1. List of polar metabolites identified in the intracellular extracts of Caki-1 cells using ^{1}H NMR spectroscopy. Resonances in bold were used for peak integration in the univariate analysis.

No.	Compound	δ ^{1}H in ppm (multiplicity, assignment)
1	Isoleucine	0.93 (t); **1.00 (d)**; 1.25 (m); 1.46 (m); 1.97 (m); 3.66 (d)
2	Leucine	0.94 (d); **0.95 (d)**; 1.70 (m); 1.69 (m); 3.73 (d)
3	Valine	**0.98 (d)**; 1.03 (d); 2.26 (m); 3.59 (d)
4	Lactate	**1.31 (d)**; 4.09 (q)
5	Alanine	**1.47 (d)**; 3.77 (q)
6	Acetate	1.90 (s)
7	Glutamate	2.04 (m); 2.11 (m); **2.34 (m)**; 3.74 (q)
8	Glutamine	2.14 (m); 2.43 (m); 3.76 (t)
9	Glutathione	2.15 (m); **2.54 (m)**; 2.94 (dd); 2.97 (dd); 3.76 (m); 4.55 (m)
10	Aspartate	**2.67 (dd)**; 2.80 (dd); 3.88 (dd)
11	Asparagine	**2.86 (dd)**; 2.94 (dd); 3.98 (q)
12	Creatine	**3.02 (s)**; 3.91 (s)
13	Phosphocreatine	**3.03 (s)**; 3.93 (s)
14	Ethanolamine	**3.13 (t)**; 3.81 (t)
15	Phosphocholine	**3.22 (s)**; 3.58 (m); 4.15 (m)
16	sn-Glycero-3-phosphocholine	**3.23 (s)**; 3.66 (m); 3.90 (m); 4.29 (m)
17	Taurine	**3.26 (t)**; 3.42 (t)
18	*Myo*-inositol	3.26 (t); 3.52 (dd); 3.61 (t); **4.05 (t)**
19	Glycine	**3.55 (s)**
20	ADP	4.22 (m); 4.38 (m); 4.61 (m); 6.15 (d); 8.28 (s); 8.54 (s)
21	ATP	4.22 (m); 4.29 (m); 4.41 (m); 4.62 (m); 6.15 (d); 8.28 (s); 8.55 (s)
22	NAD+	4.23 (m); 4.36 (m); 4,39 (m); 4.42 (m); 4.50 (m); 4.54 (m); 6.04 (d); 6.10 (d); 8.18 (s); 8.19 (t); **8.43 (s)**; 8.83 (d); 9.14 (d); 9.34 (s)
23	UDP-Glucose	3.49 (t); 3.54 (m); 3.74 (t); 3.76 (dd); 3.84 (d); 4.19 (m); 4.23 (m); 4.27 (s); 4.36 (m); 5.97 (d); 5.98 (d); 7.96 (dd)
24	UDP-Galactose	3.70 (dd); 3.74 (d); 3.90 (dd); 4.02 (dd); 4.16 (m); 4.22 (m); 4.29 (d); 5.97 (d); 7.99 (d)

Table S2. List of metabolites identified in the extracellular culture medium of Caki-1 cells by ^{1}H NMR spectroscopy. Resonances in bold were used for peak integration in the univariate analysis.

**No.**	**Compound**	**δ ^{1}H in ppm (multiplicity, assignment)**
1	Isoleucine	0.93 (t); **1.00 (d)**; 1.25 (m); 1.46 (m); 1.97 (m); 3.66 (d)
2	Leucine	0.94 (d); **0.95 (d)**; 1.70 (m); 1.69 (m); 3.73 (d)
3	Valine	**0.98 (d)**; 1.03 (d); 2.26 (m); 3.59 (d)
4	Lactate	**1.31 (d)**; 4.09 (q)
5	Alanine	**1.47 (d)**; 3.77 (q)
6	Acetate	**1.90 (s)**
7	Glutamate	2.04 (m); 2.11 (m); **2.34 (m)**; 3.74 (q)
8	Glutamine	2.14 (m); **2.43 (m)**; 3.76 (t)
10	Aspartate	2.67 (dd); **2.80 (dd)**; 3.88 (dd)
11	Asparagine	2.86 (dd); **2.94 (dd)**; 3.98 (q)
18	*Myo*-inositol	3.26 (t); 3.52 (dd); 3.61 (t); **4.05 (t)**
19	Glycine	**3.55 (s)**
25	Ethanol	1.19 (t); 3.67 (t)
26	Arginine	1.64 (m); **1.72 (m)**; 1.89 (m); 1.91 (m); 3.35 (t); 3.76 (t)
27	Methionine	2.11(m); **2.12 (s)**; 2.18 (m); 2.63 (t); 3.84 (t)
28	Pyruvate	**2.35 (s)**
29	Succinate	**2.38 (s)**
30	Pyroglutamate	2.02 (m); 2.39 (t), 2.49 (m), 4.16 (q)
31	Lysine	1.46 (m); 1.72 (m); 1.89 (m); 1.92 (m); **3.01 (t)**; 3.74 (t)
32	Glucose	3.54 (dd); 3.70 (m); 3.72 (m); 3.76 (q); 3.84 (m); 3.85 (dd); 3.91 (dd); 4.65 (d); **5.24 (d);** 3.23 (t); 3.39 (m); 3.45 (m); 3.50 (dd); 3.71 (m); 3.81(m); 3.88 (dd); 4.63 (d)
33	Fumarate	**6.50 (s)**
34	Tyrosine	**3**.05 (q); 3.18 (dd); 3.92 (m); **6.89 (d)**; 7.18 (d)
35	1-Methylhistidine	3.06 (q); 3.17 (dd); 3.68 (s); 3.96 (q); 7.05 (s); 7.76 (s)
36	Phenylalanine	3.12 (m); 3.27 (dd); 3.98 (m); 7.32 (d); 7.36 (m); 7.41 (m)
37	Formate	**8.44 (s)**

Table S3. List of lipid species identified in the intracellular extracts of Caki-1 cells by ^1H NMR spectroscopy. Resonances in bold were used for peak integration in the univariate analysis.

**No.**	**Compound**	**δ ^1H in ppm (multiplicity, assignment)**
1	Cholesterol	0.68 (s); 0.86 (d); 1.87 (d); 0.91 (d); 0.95 (m); 0.99 (m); **1.01 (s)**; 1.07 (m); 1.10 (m); 1.12 (m); 1.13 (m); 1.35 (m); 1.49 (m); 1.51 (m); 1.60 (t); 1.84 (m); 1.87 (t); 1.95 (t); 1.99 (t); 2.26 (t); 3.53 (t)
2	Cholesteryl esters	0.68 (s); 0.86 (d); 0.87 (d); 0.91 (d); 0.95 (m); 0.99 (m); **1.02 (s)**; 1.10 (m); 1.12 (m); 1.13 (m); 1.15 (m); 1.35 (m); 1.58 (m); 1.60 (m); 1.84 (t); 1.87 (t); 1.95 (t); 1.99 (t); 2.31 (t); 4.61 (m)
3	Saturated fatty acids (CH_3)	**0.88 (m)**; 1.26 (br); 1.63 (m); 2.02 (m); 2.36 (t)
4	Unsaturated fatty acids (HC=CH)	**0.88 (m)**; 1.30 (br); 1.63 (m); 2.00 (m); 2.35 (t); 2.80 (br); **5.35 (br)**
5	Phosphatidylethanolamine	**3.16 (s)**; 3.98 (br); 4.38 (br); 5.22 (m)
6	Phosphatidylcholine	**3.34 (s)**; 3.79 (br); 3.98 (br); 4.12 (m); 4.38 (br); 5.22 (br); 5.35 (br)
7	Monoglycerides	1.63 (m); 2.35 (t); **3.65 (ddd)**; 3.94 (m); 4.18(ddd)
8	Triglycerides	4.14 (dd); **4.29 (dd)**; 5.26 (t)

Table S4. List of intracellular and extracellular metabolites significantly altered in sunitinib- and pazopanib-resistant Caki-1 cells compared to parental cells.

Class/metabolite	Sunitinib-resistant Caki-1				Pazopanib-resistant Caki-1				Dysregulated metabolic pathway
	Intracellular		Extracellular		Intracellular		Extracellular		
	**ES ± SE**	***p*-value**	**ES ± SE**	***p*-value**	**ES ± SE**	***p*-value**	**ES ± SE**	***p*-value**	
Amino acids and derivatives									
Alanine	3.71 ± 1.59	0.0003	-	-	2.17 ± 1.19	0.0011	-1.32 ± 1.03 [E]	0.0207	Aminoacyl-tRNA biosynthesis
Arginine	-	-	-	-	-	-	-1.75 ± 1.11 [C]	0.0148	Aminoacyl-tRNA biosynthesis
Asparagine	-	-	-	-	-	-	-1.35 ± 1.04 [C]	0.0281	Aminoacyl-tRNA biosynthesis; alanine, aspartate, and glutamate metabolism
Aspartate	1.68 ± 1.09	0.0148	-	-	1.97 ± 1.15	0.003	1.04 ± 0.99 [C]	0.0379	Aminoacyl-tRNA biosynthesis
Glycine	1.95 ± 1.15	0.0019	-	-	1.48 ± 1.06	0.007	-2.42 ± 1.25 [C]	0.003	Purine metabolism; glycine, serine, and threonine metabolism; glutathione metabolism; aminoacyl-tRNA biosynthesis
Glutathione	2.73 ± 1.32	0.0003	-	-	-	-	-	-	Glutathione metabolism
Glutamine	-	-	-1.71 ± 1.10 [C]	0.0047	-	-	-1.32 ± 1.03 [C]	0.0281	Aminoacyl-tRNA biosynthesis; alanine, aspartate, and glutamate metabolism; purine metabolism; arginine biosynthesis
Isoleucine	2.20 ± 1.20	0.0006	-	-	2.30 ± 1.22	0.0003	-1.32 ± 1.03 [C]	0.0207	Aminoacyl-tRNA biosynthesis
Leucine	2.13 ± 1.19	0.0006	-	-	1.47 ± 1.06	0.0499	-	-	Aminoacyl-tRNA biosynthesis
Lysine	-	-	-	-	-	-	-1.35 ± 1.04 [C]	0.014	Aminoacyl-tRNA biosynthesis
Methionine	-	-	-	-	-	-	-1.25 ± 1.02 [C]	0.0193	Aminoacyl-tRNA biosynthesis
Taurine	-	-	-	-	-1.21 ± 1.02	0.0379	-	-	Aminoacyl-tRNA biosynthesis
Tyrosine	-	-	-	-	-	-	-1.11 ± 1.00 [C]	0.0148	Aminoacyl-tRNA biosynthesis
Valine	1.31 ± 1.03	0.0148	-	-	-	-	-1.32 ± 1.03 [C]	0.014	Aminoacyl-tRNA biosynthesis

Class/metabolite	Sunitinib-resistant Caki-1				Pazopanib-resistant Caki-1				Dysregulated metabolic pathway
	Intracellular		Extracellular		Intracellular		Extracellular		
	ES ± SE	*p*-value	ES ± SE	*p*-value	ES ± SE	*p*-value	ES ± SE	*p*-value	
Amino alcohols									
Ethanolamine	1.75 ± 1.11	0.0096	-	-	-	-	-	-	Glycerophospholipid metabolism
Lipid species									
Cholesterol	-	.	-	-	-2.30 ± 1.22	-	-	-	Cholesterol metabolism
Cholesteryl esters	1.31 ± 1.03	0.0260	-	-	-3.07 ± 1.41	0.0005			Cholesterol metabolism
FAs (CH_3)	-	-	-	-	-2.35 ± 1.23	0.0047	-	-	Lipid metabolism
Monoglycerides	1.30 ± 1.03	0.0281	-	-	1.70 ± 1.10	0.0207	-	-	Lipid metabolism
Phosphatidylethanolamines	1.67 ± 1.09	0.0047	-	-	-	-	-	-	Glycerophospholipid metabolism
UFAs (HC=CH)	-	-	-	-	-1.20 ± 1.02	0.0499	-	-	Lipid metabolism
Organic acids									
Acetate	-	-	-	-	-	-	-1.37 ± 1.04 [C]	0.0281	Glycolysis; gluconeogenesis; taurine and hypotaurine metabolism
Formate	-	-	-	-	-	-	-1.36 ± 1.04 [E]	0.0343	Pyruvate metabolism
Fumarate	-	-	-	-	-	-	-2.39 ± 1.24 [C]	0.0016	TCA cycle; amino acid metabolism, pyruvate metabolism
Lactate	-	-	2.31 ± 1.22 [E]	0.0019	1.19 ± 1.01	0.0281	-	-	Glycolysis; gluconeogenesis; pyruvate metabolism
Pyruvate	-	-	-	-	-	-	-1.92 ± 1.14 [E]	0.0221	Glycolysis; gluconeogenesis; TCA cycle; amino acid metabolism
Succinate	-	-	-	-	-	-	-1.51 ± 1.06 [C]	0.0221	TCA cycle; amino acid metabolism, pyruvate metabolism
Phosphocholines									

Class/metabolite	Sunitinib-resistant Caki-1				Pazopanib-resistant Caki-1				Dysregulated metabolic pathway
	Intracellular		Extracellular		Intracellular		Extracellular		
	ES ± SE	*p*-value	ES ± SE	*p*-value	ES ± SE	*p*-value	ES ± SE	*p*-value	
Phosphocholine	-1.28 ± 1.03	0.0379	-	-	-1.69 ± 1.10	0.0047	-	-	Glycerophospholipid metabolism
Glycerophosphocholine	-3.08 ± 1.41	0.0002	-	-	-3.62 ± 1.56	0.0002	-	-	Glycerophospholipid metabolism
Nucleotides									
NAD^+	-1.80 ± 1.12	0.0093	-	-	-2.31 ± 1.22	0.0011	-	-	Nicotinate and nicotinamide metabolism
Sugars									
Glucose	-	-	-	-	-	-	1.43 ± 1.05 [C]	0.0281	Glycolysis; gluconeogenesis
Myo-inositol	1.46 ± 1.06	0.0207	-	-	-	-	-	-	Galactose metabolism; inositol phosphate metabolism; phosphatidylinositol signalling system

ES: effect size, SE: standard error. Statistical significance was assessed by the Mann-Whitney test. [E, C] Metabolites excreted and consumed when compared to blanks (cell culture medium without cells).
